# Digitalization of an Industrial Process for Bearing Production

**DOI:** 10.3390/s24237783

**Published:** 2024-12-05

**Authors:** Jose-Manuel Rodriguez-Fortun, Jorge Alvarez, Luis Monzon, Ricardo Salillas, Sergio Noriega, David Escuin, David Abadia, Aitor Barrutia, Victor Gaspar, Jose Antonio Romeo, Fernando Cebrian, Rafael del-Hoyo-Alonso

**Affiliations:** 1 Technological Institute of Aragón, Calle Maria de Luna, 7-8, 50018 Zaragoza, Spain; lmonzon@ita.es (L.M.); rsalillas@ita.es (R.S.); snoriega@ita.es (S.N.); descuin@ita.es (D.E.); dabadia@ita.es (D.A.); rdelhoyo@ita.es (R.d.-H.-A.); 2 Ideko S.Coop, Member of Basque Research and Technology Alliance (BRTA), Arriaga Kalea, 2, 20870 Elgoibar, Gipuzkoa, Spain; jalvarez@ideko.es (J.A.); a.barrutia@ideko.es (A.B.); 3 Fersa Bearings, Calle Bari 37, 50197 Zaragoza, Spain; victor.gaspar@fersa.com (V.G.); joseantonio.romeo@fersa.com (J.A.R.); fernando.cebrian@fersa.com (F.C.)

**Keywords:** Industry 4.0, digitalization, waviness, burns, machine learning, grinding

## Abstract

The developments in sensing, actuation, and algorithms, both in terms of Artificial Intelligence (AI) and data treatment, have open up a wide range of possibilities for improving the quality of the production systems in diverse industrial fields. The present paper describes the automatizing process performed in a production line for high-quality bearings. The actuation considered new sensing elements at the machine level and the treatment of the information, fusing the different sources in order to detect quality defects in the grinding process (waviness, burns) and monitoring the state of the tool. At a supervision level, an AI model has been developed for monitoring the complete line and compensating deviations in the dimension of the final assembly. The project also contemplated the hardware architecture for improving the data acquisition and communication among the machines and databases, the data treatment units, and the human interfaces. The resulting system gives feedback to the operator when deviations or potential errors are detected so that the quality issues are recognized and can be amended in advance, thereby reducing the quality cost.

## 1. Introduction

The use of modern information and communication technologies in the framework of Industry 4.0 is fundamental for competitive and reliable manufacturing processes. The present work focuses on the manufacture of roll bearings, where the complex processes involved and high quality requirements can highly profit from the Industry 4.0 approach [1,2] in order to assure the extreme accuracy of the final product. The surface quality, geometric accuracy, and dimensional accuracy of manufactured bearings, as well as their surface integrity, are achieved by grinding processes. Attaining defect-free surfaces with this technology is an aspect that has been addressed through process control and optimization using various approaches such as GRINDSIM^®^ [3], GIGAS [4], or SUA [5]. However, these solutions have shown serious limitations in becoming practical tools for industrial use due to the lack of automation for acquiring and processing the necessary information to feed and calibrate the models. In order to solve this limitation, the present work presents a system for digitalizing grinding processes applied to a manufacturing line in Fersa Bearings. It uses a distributed multisource layer, merging information from different sensors in decision-making tools based on hybrid physical models and data-driven algorithms. Through this approach, defects in the process (waviness, burns), final product quality, and the state of the grinding tool for maintenance are continuously monitored and the operators are informed in real time of potential deviations.

The application of Industry 4.0 solutions are improving and optimizing the control of grinding processes by advancements in process monitoring, connectivity, data processing, and analysis capabilities [6]. The combinations of monitoring with physical simulation models and various machine learning (ML) techniques, as well as the pure application of these data-based techniques, are the most recent approaches [7]. In the present paper, two quality faults are analyzed: waviness and burns. Waviness in the contact surfaces causes operation noise, reduces the efficiency, and increases the probability of failure in loaded conditions. The analysis of data from acoustic sensors and accelerometers is used for predicting this, as proposed by [8]. The results of the statistical analysis of the information (wavelets, Fourier analysis) are the input of AI models like the ones described in [9], which compares Gaussian Mixture Models (GMM) and Hidden Markov Models (HMM) for identifying vibration faults in the raceways and the rolling element. The approach described in the present work builds up a linear frequency model based on acquired quality and accelerometer data for predicting the appearance of waviness. Apart from that, the algorithm estimates the real speed of the part for avoiding errors derived from the sliding of the workpiece in the magnetic clamp.

Burns is a thermal defect that affects the quality of the part and compromises the mechanical properties of the final product. In order to predict it, models are proposed in the literature for improving the traditional approach based on threshold evaluations. Ref. [10] trains neural networks (NN) with power consumption and Acoustic Emission (AE) data. Similarly [11] combines AE and vibration data for training. Ref. [12] extended the approach by using stacked sparse autoencoder (SSAE) with the data coming from accelerometers, AE and torque. The solution presented in the work combines physically power based models for predicting the appearance of a burn. The model uses information from the different sensors available (thermal camera, cooling fluid, wattmeter, accelerometer) and process parameters.

The tool is crucial in the quality of the grinding process and so it is important to detect damage and wear to change it before affecting the product quality. There are several approaches for monitoring the status of the tool and predicting the remaining useful life [13]. Reference [14] proposes the use of Acoustic Emission (AE) sensors. The data are treated with wavelets and the identification of the status is carried out with a Support Vector Machine. Similarly, Refs. [15,16,17] use wavelets with AE and accelerometer signals. The failure model is predicted with HMMs. The present project uses the RMS (Root Mean Square) of the AE signal, as proposed by [18,19,20] for estimating the topology of the grinding tool.

The application of ML models for monitoring production lines has attracted much interest in the last years with the advances in the technology. In this connection, some approaches have been proposed for developing ML solutions in industrial environments [21]. One important field of application is the quality control, like the work [22], where the authors tested different ML models in a PCB manufacturing line for automatic quality inspection. Another application is the recommendation of process parameters like in [23]. This is the approach in the present work, where an ML model has been developed for providing recommendations to the operators to improve the quality of the final bearing assembly.

The main contributions of this work are the development and deployment in real production conditions of local digital twins for predicting different grinding defects in real time. This permits identifying potential quality issues and applying corrective actions in advance. In addition to these, a digital twin monitors the quality of the assembly at the end of the line and supports the operators in modifying the parameters in the presence of emerging deviations. This is achieved by means of a distributed and secure architecture involving multiple information sources, sensor fusion, and sophisticated algorithms for data treatment.

The present paper is organized in five sections. A description of the line and the digitalization steps appears in Section 2.1. The algorithms are presented in Section 2.2 and the final results are in Section 3. Technical discussion is included in Section 4 and the conclusions are in Section 5. Due to privacy requirements, the units are normally not displayed in the presented results.

## 2. Materials and Methods

### 2.1. Description of the Process Line Digitalization

**Figure 1:**
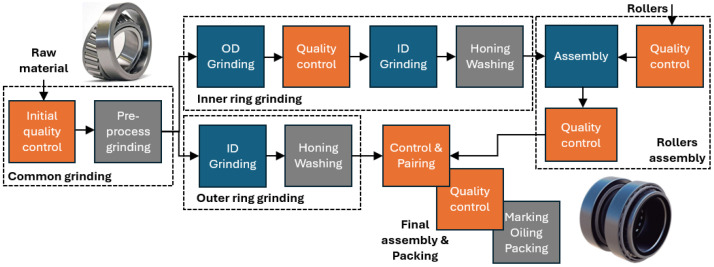
Simplified schema with the main elements of the FERSA production line.

Figure 1 shows the main elements of the Z1 production line at Fersa Bearings. It has two main process flows, corresponding with the outer and the inner rings. In both cases, the grinding process is followed by honing, washing, and quality control. After the quality control of the inner ring, the rollers are assembled and the quality of the subset is evaluated. Following that, this subset is assembled with the outer ring by pairing the parts according to their dimensions for fulfilling the highly restricted tolerances of the final product. The last processes of the final assembly are marking, oiling, and packing.

The present project is focused on the failure detection during the grinding process of the inner ring (burns and waviness), and a supervisory system of the complete line for improving the final dimension of the assembled product. In both cases, the developed system warns the operator about potential quality errors and the supervisory system also gives advice on the process parameters for assuring the quality of the final assembly.

**Figure 2:**
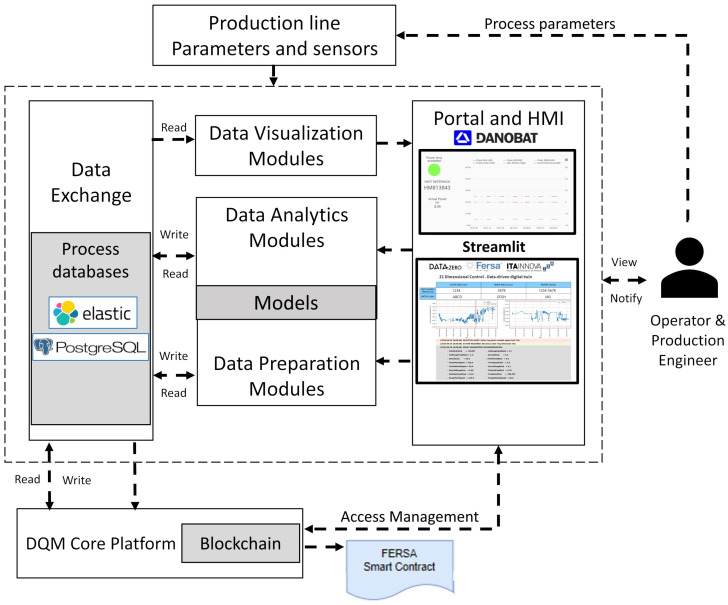
Main elements in the architecture.

Figure 2 shows the main elements in the architecture developed during the project. The information from the line, including machine sensors, quality data, and process parameters, is treated in data preparation modules and used for building behavior models in data analytics modules. The obtained information is stored in process databases connected to a DQM platform (Data Quality Management), which gathers and organizes data from the distributed multi-sensor network in order to offer adequate levels of data accuracy and precision for effective decision support and problem-solving. For strategic and sensitive data, the information is also stored in smart contracts by using blockchain technology. The data are represented in real time to the operator and process engineers via two Human Machine Interfaces (HMI) showing information in the line. On the one side, process information from the grinding machines is published using the Danobat Cloud system, which enables the visualization of acquired data and the results of waviness, burns, and tool state modules. It also serves as a notification tool for operators to take action to prevent defects generated during the grinding process. On the other side, a second HMI uses a web application with the results from the final assembly supervision model.

#### 2.1.1. New Sensors

**Figure 3:**
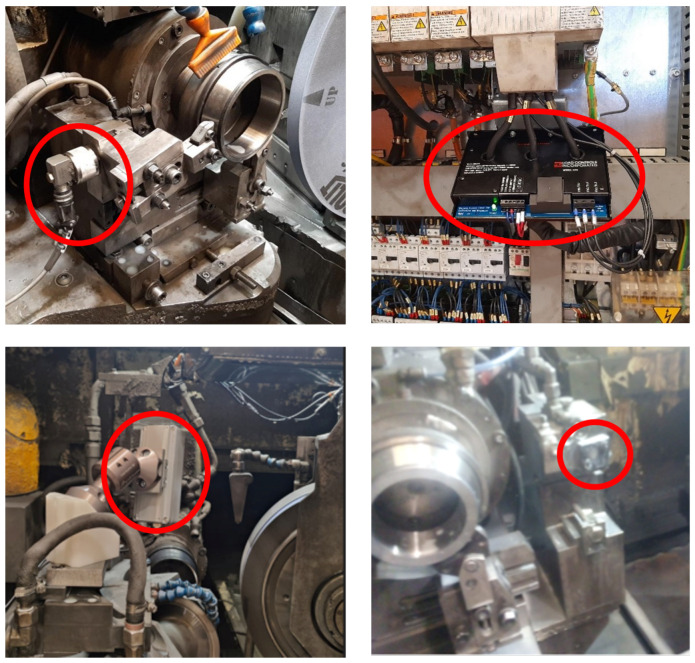
Sensors in the figure framed with a red circle: (**upper left**) accelerometer (PCB Piezotronics with a frequency range of 0.5 to 8000 Hz); (**upper right**) Equipment for grinding power acquisition; (**lower left**) thermal camera (FLIR Lepton 3.5); (**lower right**) AE sensor (Steminc 20 × 1 mm 2 Mhz R).

Several sensors (Figure 3) have been installed in the line for improving the capability of the system for predicting quality defects (waviness and burns) and the status of the tool. Waviness is related with the undulations per revolution (UPR) which characterizes the roundness profile of the bearing surface and it is affected by various types of vibrations [24], like imbalances in the rotating elements (grinding wheel, dressing actuator, workpiece) or external sources. In order to predict the risk of waviness in real time, ICP accelerometers have been installed near the rotary elements.

The burns are thermal defects caused by the high temperatures between the grinding wheel and the workpiece during the grinding process. The failure is normally evaluated offline by using eddy current technology and acid etching tests. In order to have an online prediction of the thermal damage, a virtual sensor is proposed to estimate the grinding conditions when the temperature of the grinding area approaches the limit of workpiece thermal damage. This virtual sensor uses a power meter connected to the three-phase spindle motor, a coolant sensor, and a thermal camera.

For the state of the tooling, an acoustic emission sensor has been installed at the dressing tool column.

#### 2.1.2. Communication Hardware

The information from the different sensors at the grinding machines is transmitted to the Danobat box system, which is used for data collection, analytics, and communication. Its compatibility with different communication protocols simplifies the integration with the different components. It connects the Danobat Cloud with the CNC (Computer Numerical Control) local machine and the process databases. The communication protocol from the new sensors to the Danobat box depends on the information source. OPC-UA is used for coolant concentration acquisition, while MODBUS is used for pre-processed data from accelerometers (10 kHz) and power meters coming from the installed PAC system (Programmable Automation Controller) INGESYS IC3. The high rate information from the AE sensor is acquired by a STEMlab system at 1 MHz and processed in a separate unit connected to the Danobat box using Ethernet. This is also the case for the analysis of the information from the thermal camera, which is connected via USB to a controller, which afterwards sends the information to the Danobat box using Ethernet. The CNC parameters of the grinding machines are collected using specific libraries developed in C# that allow access to the data via the machine manufacturer’s .DLL files.

For the analysis of the line, a dedicated server connects to the FERSA databases to retrieve information from the manufacturing machines (ElasticSearch database) and the quality inspection machine results (PostgreSQL). The information is treated in a Python model of the line and the results are published in the line HMI with a web application based on Streamlit.

#### 2.1.3. Blockchain

**Figure 4:**
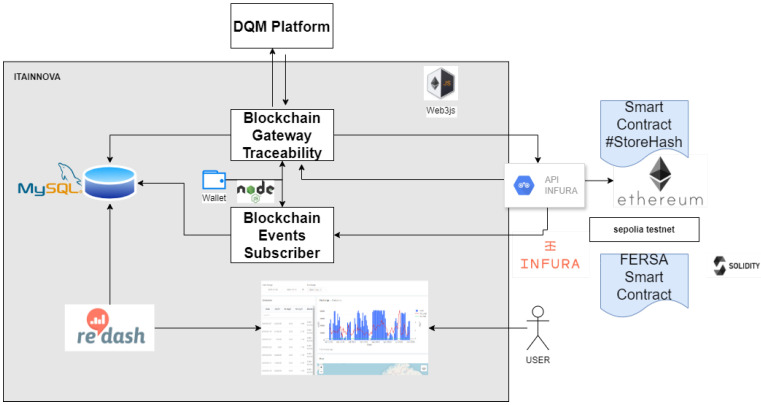
Description of the traceability blockchain architecture.

The most sensitive data can be protected by using blockchain thanks to the digitalization of the line. This is a practice that has been used in other fields, like, for example, in [25], where blockchain assured the traceability of data sets in the mining industry. This technology has been implemented in the project for proving its potential in sharing critical quality results with customers and suppliers. The system acquires the critical information from a production batch, obtaining its digital fingerprint and storing it in blockchain. The system can be configured to acquire different quality results and process parameters from the DQM platform. Once implemented, the cost of its configuration and adaptation to different production conditions is negligible. Figure 4 depicts the blockchain architecture and its components for traceability and data management. The traceability is assured through the hashing process. This process involves a one-way cryptographic function that generates a consistent digital fingerprint of a certain length from any given dataset. Storing the hash of the data on a blockchain ensures its immutability. However, the hash does not reveal any original data information, as it cannot be reversed. The hashing outcome at any point can be compared with the historical hash recorded when the dataset was first created.

The blockchain gateway is responsible for distributing the information between the different modules. The smart contract with the original information and the hash (SHA256) are obtained using Ethereum. The resulting hash is stored in a MySQL database and it can be visualized by using Redash. The wallet contains the private key to execute reliable transactions in Ethereum Testnet and to confirm the ones coming from suppliers or customers interested in data sharing.

### 2.2. Description of the Grinding Quality Prediction Algorithms

In this section, the grinding quality prediction algorithms running at machine level are described. These algorithms are focused on the identification of grinding failures (waviness and burns) and the evaluation of the grinding tool surface.

#### 2.2.1. Waviness Prediction Algorithm

**Figure 5:**
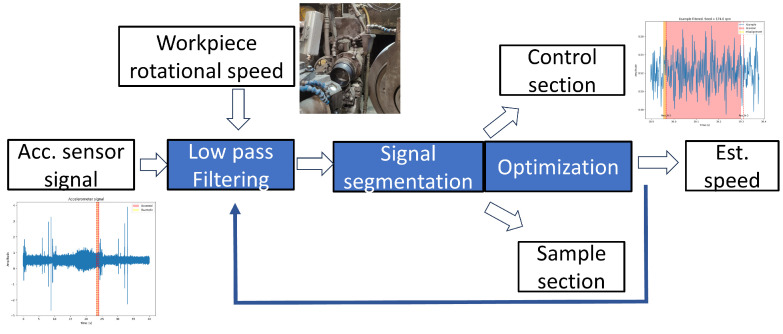
Speed algorithm global architecture.

The waviness algorithm predicts the appearance of this failure in the current workpiece by using the information from the accelerometers near the machined part. This prediction is performed in two phases: firstly, the turning speed of the part is estimated; and, secondly, the presence of waviness is evaluated. The estimation of the turning speed is necessary due to the slippage of the workpiece on the magnetic clamp, resulting in a discrepancy between the commanded velocity and the actual one. To fix this error, the algorithm in Figure 5 is used for estimating the real speed Ω (rad/s). First of all, the accelerometer signal is divided into sections, each corresponding to one wheel revolution. After that, the algorithm compares a control section that represents the most recent revolution with a sample section from the previous revolutions. The estimation of the speed is obtained by maximizing the correlation between both sections.

With the refined workpiece speed, the accelerometer signal $x_{acc}\left(t\right)$ is again split into individual revolutions for evaluating the appearance of waviness. The analysis uses the signal sections corresponding with the fine grinding and chirp phases, as the surface is closer to the final result. After being filtered, the FFT of the signal $X_{acc}\left(w\right)$ is calculated. The frequency scale *w* (rad/s) is converted to the UPR scale by the expression below:

(1) $$UPR=\frac{w}{\Omega }$$

**Figure 6:**
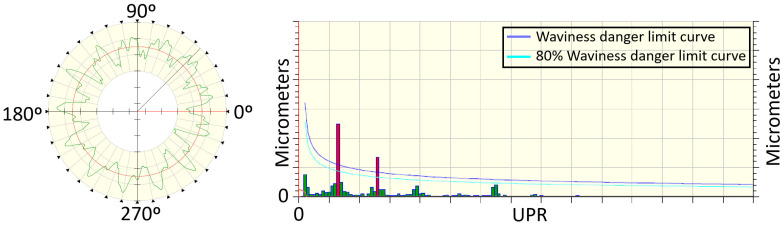
Post-manufacturing quality process result for waviness analysis. Purple line shows the threshold harmonic content for the waviness appearance. Light blue line shows 80% probability threshold for waviness appearance.

The prediction of the waviness measurement $\hat{X}\left(UPR\right)$ (Figure 6) is performed by scaling the values $X_{acc}\left(UPR\right)$ with a discrete gain model $F_{s,i}$:

(2) $$\hat{X}\left(i\right)=X_{acc}\left(i\right)\cdot F_{s,i}$$

for each $i=1\ldots UPR_{max}$.

The values $F_{s,i}$ have been identified by an offline process correlating quality measurements with the accelerometer signals in different parts. The result of this identification is a set of gain models. The probability of waviness is estimated by evaluating the number of models that predict values over the quality limit (Figure 6).

#### 2.2.2. Thermal Damage Prediction Algorithm

For the thermal damage prediction, the approach uses models based on the theoretical development by Jaeger and its application by Malkin [26], which rely on estimating maximum temperature as the sole indicator [27,28]. This concept is based on the relationship between the surface’s thermal damage-free temperature limit $\theta_{m}$ and the grinding power *P* required to reach that temperature:

(3) $$\theta_{m}=\frac{\beta \alpha_{w}^{1/2}\epsilon P}{k_{w}bd_{e}^{1/4}v_{w}^{1/2}a^{1/4}}$$

**Figure 7:**
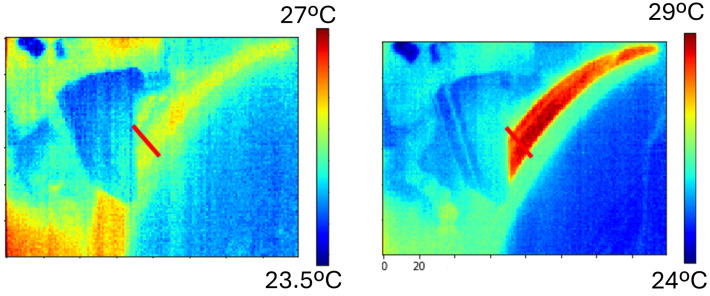
Thermal image obtained during the grinding process.

Other parameters influencing the calculation include the thermal parameters of the workpiece, such as the thermal conductivity $k_{w}$ and thermal diffusivity $\alpha_{w}$ of the material, the equivalent diameter $d_{e}$, the grinding width *b*, and the grinding conditions corresponding to the workpiece speed $v_{w}$ and the depth of cut *a*, as well as the constant dependent on the considered shape of the energy source β. One critical parameter in the calculation is the energy partition ϵ, typically obtained through experimentation. The use of a thermal camera permits approaching it by estimating the power used for heating the workpiece and the tool from the measured temperature (Figure 7).

This approach has been adapted to the case of bearing grinding, where different bearing surfaces are ground on each machine, requiring a specific formulation in each case.

**Figure 8:**
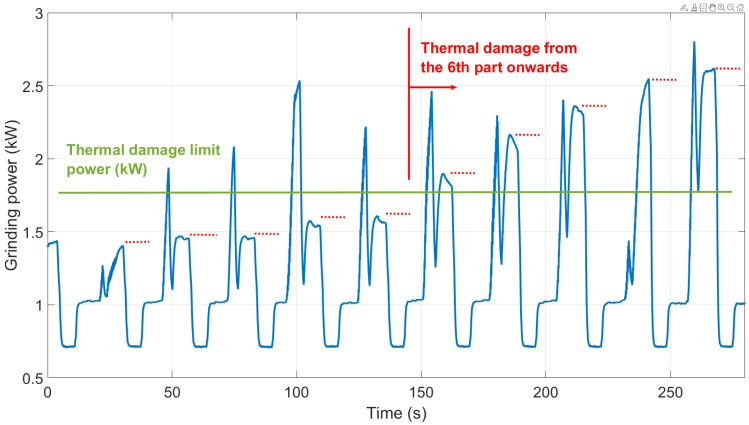
Schematic example of the virtual sensor solution for thermal damage prediction based on the grinding power measurement and the calculation of the limiting value.

Once the power limit calculations are developed, the solution of the predictive model relies on comparing the consumed power with the calculated limit. If the grinding power exceeds it on a particular workpiece, there is a risk of thermal damage occurrence. Figure 8 shows a schematic example of the power evolution of different workpieces over time. The red dots are the sampled measurements from the complete measured power signal (blue line). These values are then compared with the power limit (green line). In the case described in the figure, thermal damage would occur after the sixth piece.

#### 2.2.3. Tool State Identification Algorithm

This algorithm is intended to identify variations in the surface of the grinding tool. Given its influence in the quality of the process, its state is evaluated after each dressing in order to detect deviations in its topology. For this, AE sensors are employed to acquire high-frequency patterns. The approach follows the works from Oliveira in [18,19]. The sensor is placed near the diamond tool used for the dressing process, which has point contact with the wheel surface.

The data collected from the acoustic emission sensor is acquired at very high frequency to identify surface effects at grain level. This high acquisition rate generates a vast amount of raw data, which demands considerable computational resources to process and convert them into meaningful information. In consequence, this process is carried out offline after the data acquisition. The signal is treated using a high-pass filter to remove the low-frequency components. This step is critical to enhance the clarity of the data so that only the most relevant high-frequency components are analyzed.

**Figure 9:**
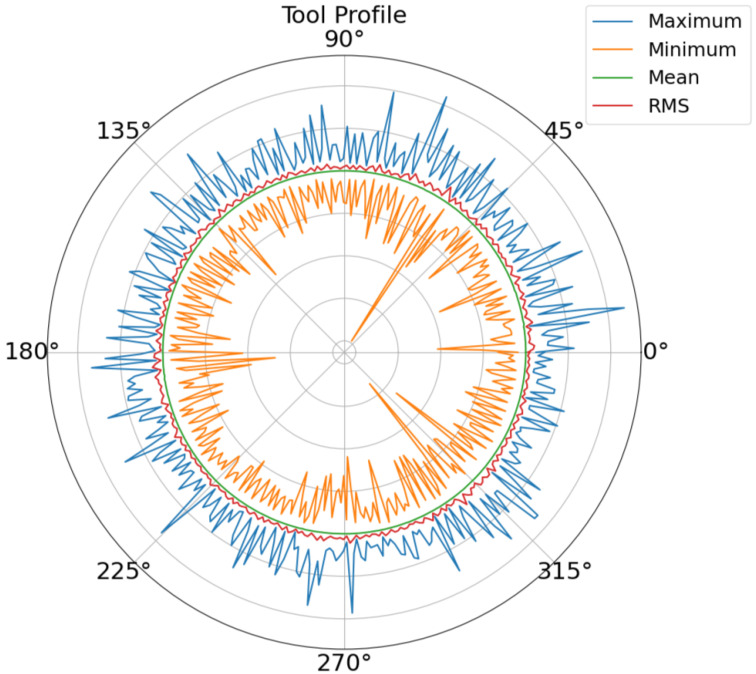
Tool profile estimation with maximum, minimum, mean, and RMS values.

**Figure 10:**
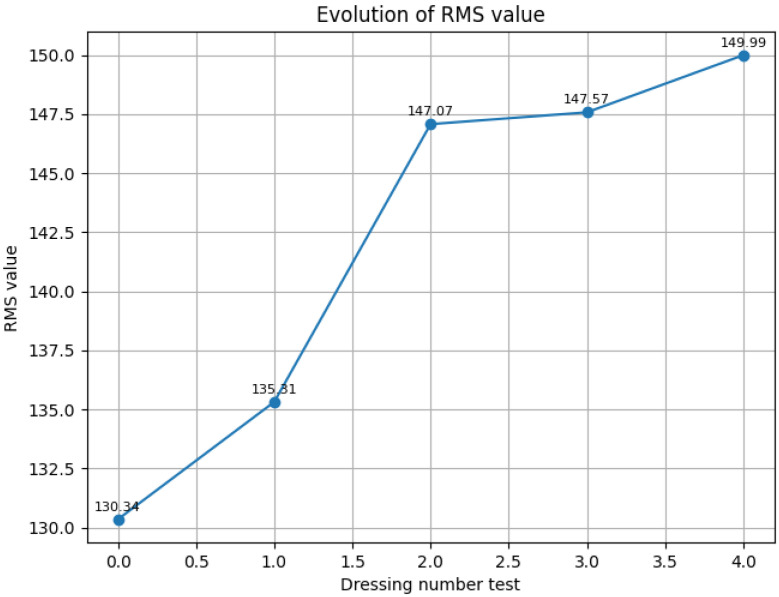
Evolution of RMS value over consecutive dressing operations.

Once the noise has been filtered out, the cleaned signal is used for different analyses in the frequency and the time domains. The resulting signal is divided into sections for each revolution of the wheel. With this information, the tool surface is estimated using the values from the last rotations of the dressing process by calculating RMS, mean, maximum, and minimum AE values at specific angles (Figure 9). These values are tracked to estimate the wear of the tool or the appearance of surface defects. Apart from topological representations, the RMS value of the final turn is also used as a numerical indicator of the tool state. Figure 10 shows the increase in this value over consecutive dressings. This test was performed after several machining processes without dressing. After that, the effect of consecutive dressings can be seen in the evolution of the RMS value as the tool grain is recovered. From that test, a threshold RMS value of 142 was identified for the tool surface quality.

### 2.3. Assembly Quality Prediction

**Figure 11:**
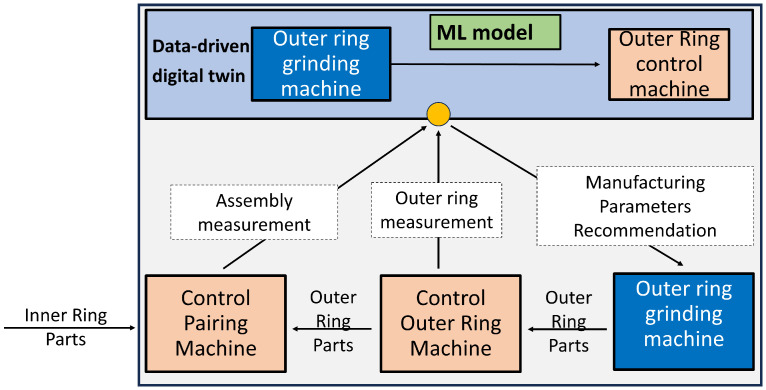
Line dimensional control.

In this section, the assembly quality supervisory algorithm at complete line level is described. The main purpose of this solution is assuring the dimensions of the final assembly. It uses the information from the existing quality inspection machines in the production line, the different machine CNCs, and the sensors previously described. Therefore, a data-driven digital twin has been developed to estimate the required outer ring dimension for fulfilling the tolerance of the final assembly, considering all components (inner ring, outer ring, rollers). The optimization algorithm is described in Figure 11. The dimensions of the outer ring and the final assembly are continuously measured at the end of the line. If they do not meet the specified tolerances, the data-driven digital twin is consulted and it provides recommendations for manufacturing parameters in the grinding machines of the outer ring (Figure 1).

In the following, the phases for developing the machine learning model are described.

#### 2.3.1. Feature Extraction & Feature Engineering

The sources of the data are:

- CNC: Manufacturing configuration parameters comprising 79 variables with a sampling frequency of 5–8 s. These parameters are measurement once per part.
- IC2: 39 accelerometer variables sampled at 1 Hz.
- IC3: 4 power consumption measurements sampled at 1 Hz.

Since the different groups of variables have different sampling frequencies, it was decided to standardize all variables to the CNC frequency, which coincides with the production of each part. During the analysis, some variables were removed from the data flow when they were either informative or constant. In addition to that, derived variables are also obtained by applying PCA (Principal Component Analysis) and Univariate linear regression relating each variable with the height of the outer ring. Finally, all generated variables are normalized using a standard scaler to have zero mean and unit standard deviation.

#### 2.3.2. Feature Selection

Once the candidate variables have been generated for the machine learning models, a feature selection process is carried out. The selected technique has been Recursive Feature Elimination with Cross-Validation (RFECV), which is a method used with models like Random Forest for automatic feature selection. It starts with all features, evaluates their importance through cross-validation, and iteratively removes the least important ones until the optimal performance is achieved. The RFECV model has been trained with all candidate variables to predict the outer ring dimension. The result of the analysis showed that the CNC production variables are the most important when manufacturing the outer rings.

#### 2.3.3. Data Preparation

From the selected variables, two subsets are distinguished: High-Variability Variables (HVV) (grinding tool diameter, final advance of the tool), which take different values for each successive part; and Low-Variability Variables (LVV), which remain constant during entire production runs and their value is 0 or 1. The input to the system is a set of the previous variables.

#### 2.3.4. Modeling

The model training had the following characteristics:

- Five types of machine learning models were trained: Multi-Layer Perceptron, Decision Tree, Random Forest, SVM, and Gradient Boosting;
- For each model, a hyper-parameter-tuning process was conducted using Bayesian optimization methods;
- Model evaluation was performed using cross-validation techniques, using the $R^{2}$ metric as the selection criterion.

The selected model is a Random Forest with a minimum leaf node size of 3 observations, 190 trees, and no limit on the maximum depth of the tree.

## 3. Results

This section summarizes the results obtained with the previous algorithms.

### 3.1. Waviness

**Figure 12:**
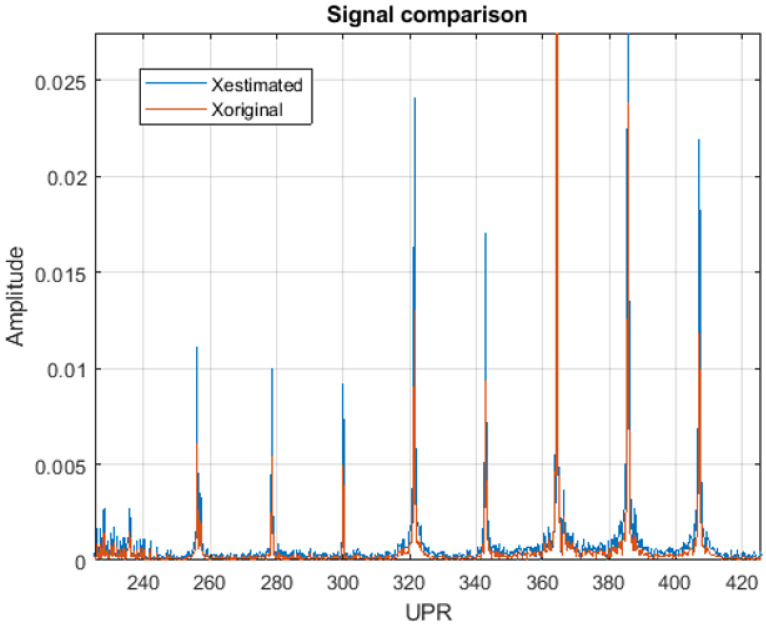
Comparison between the predicted harmonic content and the result from the offline quality control.

**Figure 13:**
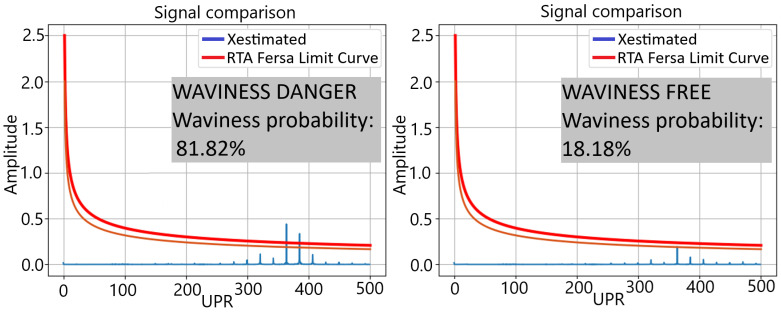
Comparison of the predicted harmonic content and the limits of the waviness protocol.

Several test batches have been conducted at Fersa Bearings facilities to validate the waviness prediction algorithm. For this, the operation conditions were changed in order to force the appearance of defects. The comparison of the predicted signals and the measured ones can be observed in Figure 12. Figure 13 shows the predicted quality result obtained with two different parts. As a result of the algorithm, which is shown in the HMI, the operator should consider modifying the grinding parameters or dressing the tool to reduce the probability of waviness in the case of receiving a warning notification. To make that decision, the operator also has the information from the tool state algorithm (Figure 9 and Figure 10).

### 3.2. Burns

**Figure 14:**
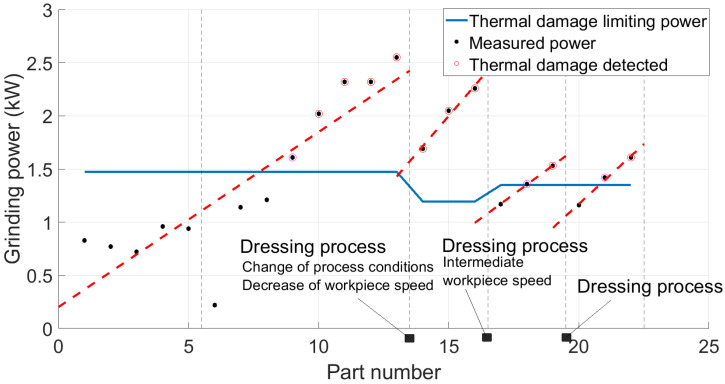
Results of thermal damage for different tests and validation of the prediction tool. The blue line represents the thermal limit and the dots represent the measured power. The circled dots show the workpieces that presented burns during the quality control.

A series of tests have been conducted on the different grinding processes, where different batches were processed without intermediate dressing. The grinding power was acquired during the process. Apart from that, controlled variations were made to evaluate the influence of other parameters like workpiece rotational speed or feed rate. After that, the ground surfaces were inspected using acid baths or visual inspection. Figure 14 shows the power results for each ground workpiece and their trend over time. It was observed that, once the power exceeded the predicted limit in each series, the workpieces exhibited thermal damage, thereby validating the predictive model for different grinding conditions.

### 3.3. Line Dimensional Control

**Figure 15:**
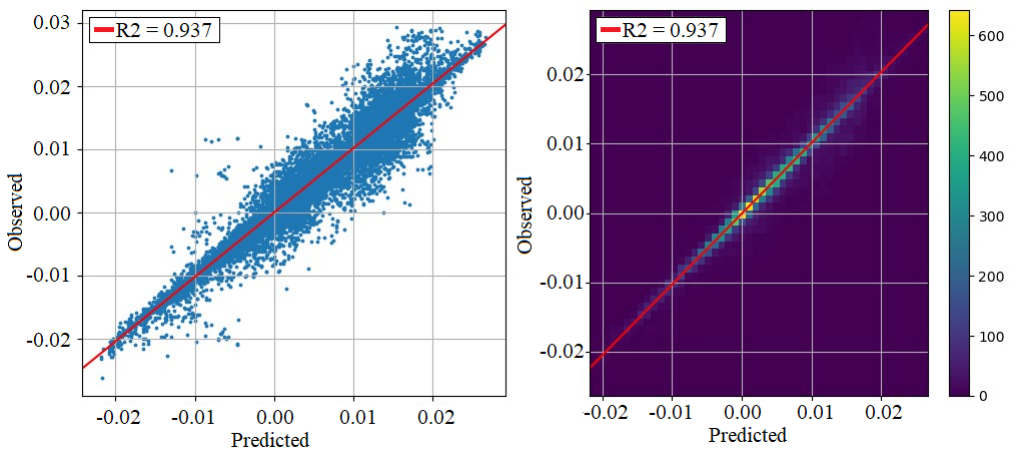
Values predicted by the dimensional line model versus actual values measured on the control machine. Scatter plot (**left**); 2D histogram (**right**).

**Figure 16:**
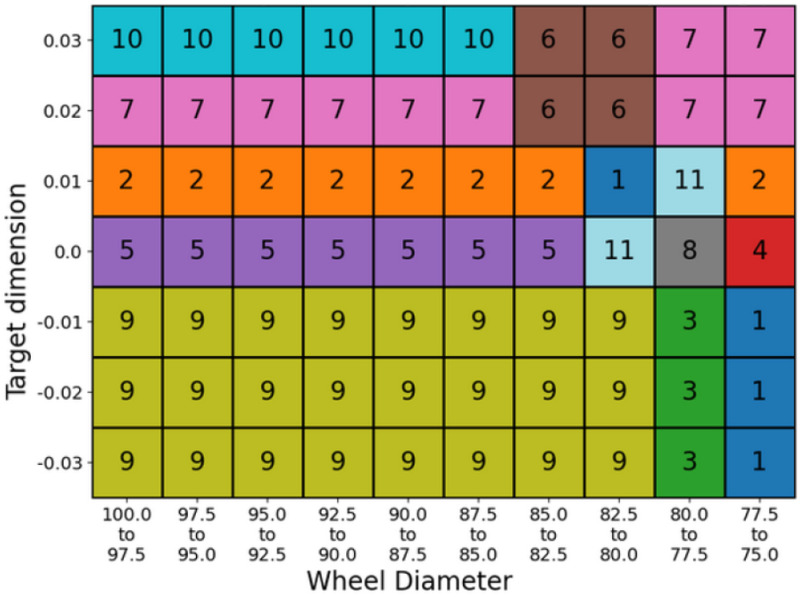
Recommended production pattern based on operating point (each number/color represents a production pattern). Units are not displayed due to Fersa’s privacy policy.

The machine learning model that constitutes the data-driven digital twin achieves an $R^{2}$ above 0.93, as shown in Figure 15. Figure 16 shows that the dimensional model recommends different production patterns (a set of manufacturing parameters for the grinding machine of the outer ring) adapted to the operating point—in this case, the grinding wheel diameter.

### 3.4. Data Visualization

**Figure 17:**
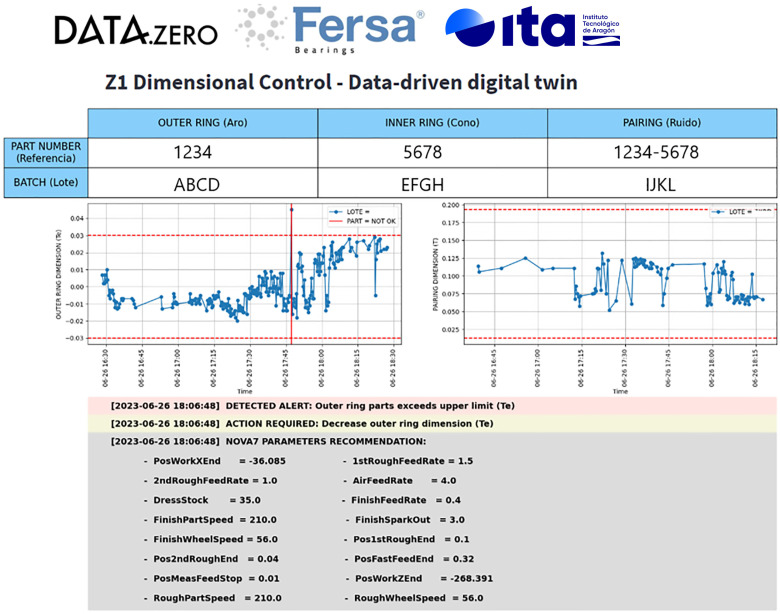
Example of the interface for the assembly quality control.

**Figure 18:**
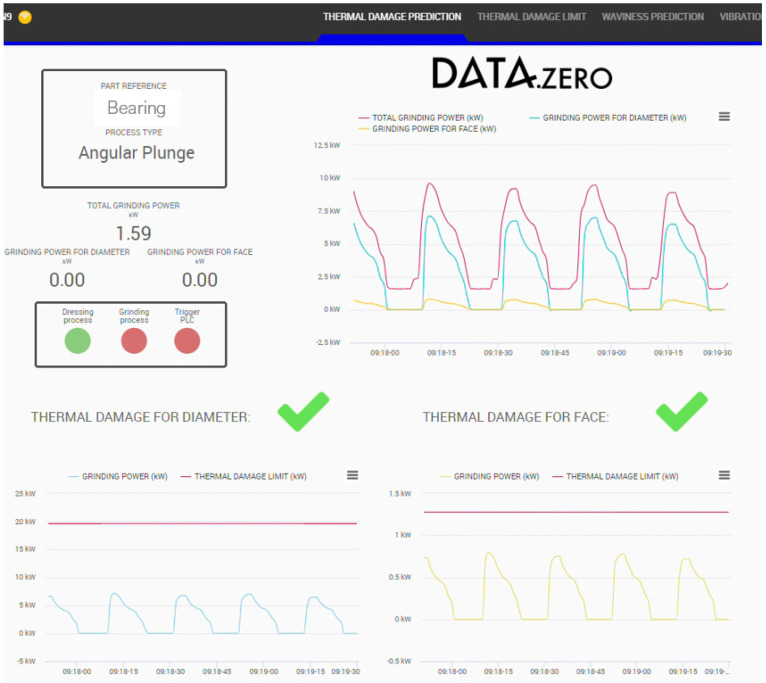
Example of the interface for the grinding quality control.

All the information generated is visualized in two different HMIs placed at the line: one for the assembly quality (Figure 17) and another one for the grinding quality (Figure 18).

## 4. Discussion

Throughout the project, the Z1 line at Fersa Bearings was equipped with sensors and networked computation units at machine and line levels. After that, different tests have been carried out in real production conditions for evaluating the implemented algorithms, whose results are presented in Section 3.

The algorithm for estimating the waviness using the information from the accelerometers has shown good results. The Figure 12 compares the quality control of the part surface obtained in the laboratory (Xoriginal) with the predicted one using sensor data during the grinding process (Xestimated). The figure represents the spatial frequency distribution in a machined inner ring. The quality of the part requires that this distribution lays below certain threshold curve fixed in the quality norm provided by the customer OEMs (Original Equipment Manufacturer). The Figure 6 shows the result in two different samples, one fulfilling the restriction and another one laying out of tolerance. The possibility of predicting this result using online accelerometer data permits detecting the failure much faster than only using sampled laboratory measurements.

With regard to the thermal damage, the presented algorithm has been tested in different conditions until exceeding the power limits for the appearance of burns. This can be seen in Figure 14, where the conditions were modified with the logical change in the power threshold (blue line). During the first run, the thermal damage starts appearing in the ninth part after dressing. After that, the decrease in the workpiece speed causes a reduction in the power limits, and the thermal damage is already present in the first part. Using an intermediate workpiece speed and following the same procedure, results show that the thermal damage appears in the second or third part. This influence of the workpiece speed was already analyzed in previous research [28], but under controlled conditions in a lab environment. In this case, the prediction model is able to accurately predict the thermal behavior under industrial conditions with slight differences coming from changes in process parameters. It was observed in every case that, once the power exceeded the predicted limit in each series, the workpieces exhibited thermal damage when tested in the laboratory thereby validating the predictive model for different grinding conditions.

The tool state identification algorithm tracks the evolution in the surface of the tool by analyzing the high-frequency acoustic emission signals collected during the dressing process. By applying a high-pass filter to isolate relevant high-frequency information and selecting key features such as RMS evolution and FFT, the algorithm monitors changes in the tool’s surface. For example, Figure 10 shows that the AE RMS value increases in consecutive dressing operations showing a correlation with the surface state. This analysis will be extended in future activities.

The previous results were related with the appearance of machining defects by using the machine local estimating algorithms. The line also has a quality supervisory algorithm for the quality of the final assembly. Figure 15 shows the capability of the developed data-driven digital twin model to predict the outer ring height dimension, which is critical for the bearing assembly. The coefficient of determination obtained by the model ($R^{2}$ = 0.937) shows a high linear correlation between the predicted and the observed (measured) value of the outer ring height dimension. By taking into account the real time quality value measured in the quality inspection machines (outer ring and assembly dimensions) the supervisory algorithm proposes an optimal set of parameters, according to the current operating point, for obtaining the required dimension in the assembly. In this way, actions performed by the model (prediction and recommendation) define an operator support system for improving the assembly process. An example of the model recommendations, given a specific operating point, is shown in Figure 16.

Although this article focuses on a digitalization solution tailored to grinding processes applied to small-sized bearings in the automotive sector, this solution is easily scalable to other grinding processes and sectors. The architecture can be directly adapted to other manufacturing lines, as the equipment is designed to work with various numerical controls, and the integration of sensors is similar across different grinding machines. In all cases, a similar hardware structure is possible. First, local sensors at the machine level are connected to edge computing devices for fast evaluations (data treatment, virtual sensors). Then, communication networks link those local elements to data lakes, receiving all information and feeding it to ML models, which support the activity of the operator and process engineer. The interaction with the human operator is assured by one or several HMIs. Likewise, the ML models can be deployed for other grinding applications, as they are properly parameterized based on part data (such as geometry or material), tool data, and process parameters. This approach enables the digitalization of manufacturing lines for large bearings in sectors like energy, as well as other types of components in the automotive sector, such as drive shafts or rotor shafts for electric vehicles.

## 5. Conclusions

The present work describes the activities for digitalizing the Z1 line at Fersa Bearings. The main contributions are twofold. On the one side, it presents the complete digitalization of a real production line with hardware (sensors, control units, communication modules, and HMI) and software elements (data treatment, observers, and models) to improve the knowledge of the process in real time and to take measures when deviations are detected. On the other side, it presents the models and algorithms for predicting grinding failures (waviness and burns) and estimating the tool state at the machine level, combined with the supervisory system at the line level, recommending process parameters for assuring the dimensions of the complete assembly. Once the digitalization of the line has been achieved, the complete system has been tested in real operation conditions.

Once the line has been digitalized, the quantity of data available is much larger and it is possible to improve the existing algorithms using the available components. In the short term, the main future actions will be focused on improving the available models with more experimental data and tuning them for several part references, as the current results have been obtained for a single part number. With regard to the blockchain components, it is under analysis to extend it to data spaces from the automotive industry for data exchange between suppliers and OEMs.

## Data Availability

The data are not publicly available due to privacy or ethical restrictions.
